# Constitutional 2p16.3 deletion including *MSH6* and *FBXO11* in a boy with developmental delay and diffuse large B-cell lymphoma

**DOI:** 10.1007/s10689-021-00244-2

**Published:** 2021-04-03

**Authors:** N. van Engelen, F. van Dijk, E. Waanders, A. Buijs, M. A. Vermeulen, J. L. C. Loeffen, R. P. Kuiper, M. C. J. Jongmans

**Affiliations:** 1 Princess Máxima Center for Pediatric Oncology, Utrecht, The Netherlands; 2 Department of Genetics, University Medical Center Utrecht, Utrecht, The Netherlands

**Keywords:** FBXO11, DLBCL (diffuse large B-cell lymphoma), Developmental delay, MSH6, BCL6

## Abstract

We describe a case of a boy with neurodevelopmental delay and a diffuse large B-cell lymphoma (DLBCL) in whom we discovered a germline de novo 2p16.3 deletion including *MSH6* and part of the *FBXO11* gene. A causative role for *MSH6* in cancer development was excluded based on tumor characteristics. The constitutional *FBXO11* deletion explains the neurodevelopmental delay in the patient. The FBXO11 protein is involved in BCL-6 ubiquitination and BCL-6 is required for the germinal center reaction resulting in B cell differentiation. Somatic loss of function alterations of *FBXO11* result in BCL-6 overexpression which is a known driver in DLBCL. We therefore consider that a causative relationship between the germline *FBXO11* deletion and the development of DLBCL in this boy is conceivable.

**Supplementary Information:**

The online version contains supplementary material available at 10.1007/s10689-021-00244-2.

## **Introduction**

Cancer in childhood is mostly sporadic, but in an estimated 6–10%, a genetic predisposition plays an important role [1–3]. Many of the children with underlying germline mutations have additional syndromic features, but the exact proportion of children with a cancer predisposition syndrome (CPS) is unknown. The identification of novel predisposing genes is an ongoing process [4, 5].

It is often challenging to discover or confirm the causal link between a (new) germline pathogenic variant in a gene and tumor development in an individual. Especially rare genetic syndromes with low penetrance for cancer are difficult to identify as a CPS. Examples are Weaver syndrome, caused by pathogenic variants in *EZH2*, and the Diets-Jongmans syndrome, caused by pathogenic variants in *KDM3B* [6–9]. Large case series are necessary to provide sufficient evidence that these patients indeed have an increased risk for tumor development. However, a first suspicion of a causative correlation often starts with cancer in a single case combined with knowledge of somatic cancer driving alterations in the gene of interest.

In this case report we describe a boy with developmental delay and a diffuse large B-cell lymphoma in whom we identified a de novo germline 2p16.3 deletion (134.1 kb), including *MSH6* and part of *FBXO11*. The developmental delay can be explained by the *FBXO11* deletion [10, 11]. We interrogate the role of this germline deletion in cancer development in this boy.

## Case report

The patient is a 5-year-old boy who was born after an uncomplicated pregnancy and delivery. His parents are a healthy, non-consanguineous couple. The family history was unremarkable. The patient had a congenital tooth which was extracted at the age of 4 years. His cognitive and speech development are severely delayed for which he attends special education. The boy’s behavior is characterized by hyperactivity and a short attention span. His vision and hearing are normal. He had no history of frequent or severe infections. Physical examination showed normal height and head circumference for age. He has several facial dysmorphisms including a wide forehead, wide palpebral fissures, long eyelashes and a thin upper lip. He has no hyper- nor hypopigmentation of the skin and normal extremities.

**Fig. 1:**
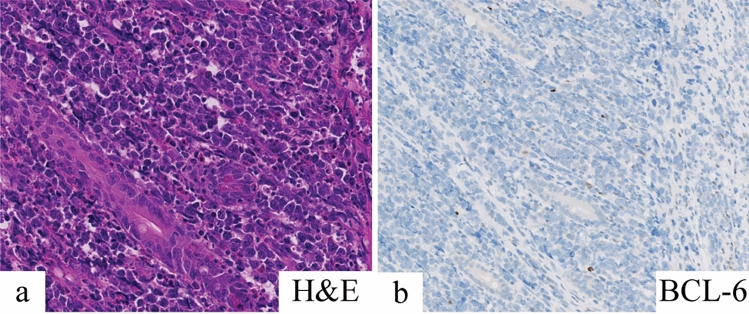
Microscopic image of the DLBCL showing the tumor cells between the crypts in the lamina propria. **a** Hematoxylin and eosin (H&E) staining was used on the tissue. **b** The tumor cells were stained with BCL-6, showing loss of BCL-6 expression in the tumor of the patient

At the age of 5 years, the patient was referred to the hospital with pain in the lower right quadrant of the abdomen for 2.5 weeks that had worsened over time. Additional symptoms were frequent nosebleeds, easy bruising and weight loss. Physical examination revealed an enlarged spleen and liver. Laboratory tests showed hemoglobin 5.5 mmol/L, thrombocytes 36 × 10^9^/L, leukocytes 67.8 × 10^9^/L, ASAT 159 U/L, ALAT 20 U/L, LD 10,701 U/L, urine acid 0.9 mmol/L, CRP 68 mg/L. An ultrasound revealed hepatosplenomegaly and an ileocaecal intussusception. Conventional imaging showed no signs of a lymphoma. A hydrostatic reposition was performed but was insufficient. The next day, an ileal resection with primary anastomosis was performed. Evaluation of the resection material by the pathologist revealed a BCL-2 positive and BCL-6 negative high-grade B-cell lymphoma of the small intestine (Fig. 1). Based on morphology and BCL-6 negativity the preferential diagnosis was a DLBCL above a Burkitt lymphoma. Additional examination showed 72% blasts in the bone marrow and 3% blasts in the cerebrospinal fluid. Using FISH analysis, a complex t(8;14) IGH-MYC translocation was detected, but no translocations of the *BCL2* and *BCL6* genes were found. Immunohistochemistry for the mismatch repair proteins (MLH1, MSH2, MSH6, PMS2) was positive, and the tumor showed no microsatellite instability (BAT25, BAT26, BAT40, D17S250, D5S346, D2S123 markers).

Considering the typical morphological and immunohistochemical aspect of the tumor, the diagnosis of a cMYC-IGH translocated diffuse large B-cell lymphoma (DLBCL) with bone marrow and central nervous system involvement was made. The patient was subsequently treated according to the EICNHL inter-B-NHL Ritux 2010 protocol (treatment arm C3). The treatment was complicated by multiple episodes of paralytic ileus and steroid induced diabetes mellitus. The patient finished his treatment according to the protocol and showed no sign of relapsed disease or long-term sequelae of treatment 1.5 years post treatment.

**Fig. 2:**
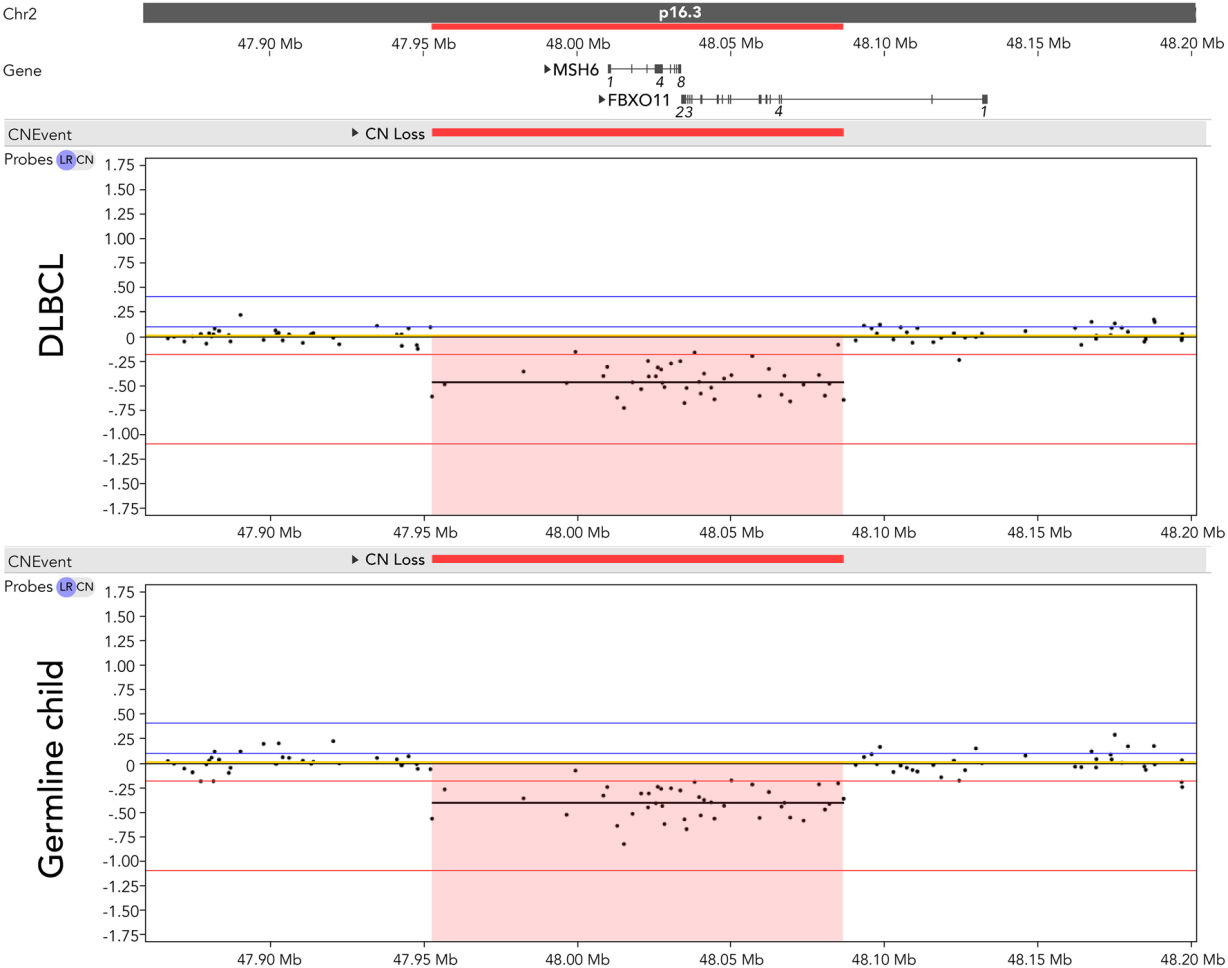
Illumina CytoSNP-850K SNP-array analyses revealed a de novo 134-kb 2p16.3 deletion including the *MSH6* gene and the 3′ prime part of the *FBXO11* gene in both DLBCL and germline DNA of the child. DLBCL DNA was derived from bone marrow at diagnosis. Germline DNA was derived from blood in remission

As part of the diagnostic process for DLBCL, a CytoSNP-850 K BeadChip SNP array (Illumina, San Diego, CA, USA) on lymphoma derived DNA was performed. This revealed a 134-kb deletion in 2p16.3 including the *MSH6* gene and exons 2–23 of the *FBXO11* gene (Fig. 2), and a copy-number-neutral loss-of-heterozygosity of part of the short arm of chromosome 17 (pter-p12) (not shown), suggesting a 17p13.1 *TP53* mutation (see below). Because of the combination of developmental delay and DLBCL the patient was referred to a clinical geneticist for further evaluation. The 2p16.3 deletion was also detected in a remission sample from blood by SNP-array and therefore marked as a germline deletion. The deletion of *MSH6* was confirmed by Multiplex Ligation-dependent Probe Amplification (MLPA). A second germline *MSH6* variant was not detected using Sanger Sequencing, ruling out Constitutional Mismatch Repair Deficiency (CMMRD) underlying the DLBCL. Both parents tested negative for the deletion by SNP array analyses on peripheral blood DNA. Whole exome sequencing was performed on DNA from the tumor and a germline sample derived from blood. Analysis of somatic variants revealed a *TP53* hotspot variant: c.742G > A (p.R248W) and several additional aberrations (Supplementary Table 1). No somatic alterations in *FBXO11*, *MSH6* or *BCL6* were present, nor did we find hypermutation in the tumor or a mutational signature related to MMR deficiency.

## Discussion

In this case report we describe a child with developmental delay who developed a diffuse large B-cell lymphoma. Genetic testing revealed a de novo germline 2p16.3 deletion including the *MSH6* gene and a large part of the *FBXO11* gene.

*MSH6* or mutS homolog 6 encodes for the DNA mismatch repair (MMR) protein MSH6, a member of the Mutator S family. Germline heterozygous loss-of-function variants in the MMR genes, *MHL1*, *MSH2*, *MHS6*, and *PMS2* are associated with autosomal dominant Lynch syndrome (OMIM# 120435), which is associated with an increased risk of predominantly colorectal cancer and endometrial cancer in adults at a young age [12]. DLBCL is not a recurrent feature of Lynch syndrome, but DLBCL with microsatellite instability was reported in a patient with a germline *MLH1* mutation [13]. Biallelic pathogenic germline variants in the MMR genes result in Constitutional Mismatch Repair Deficiency (CMMRD, OMIM# 276300) [14]. CMMRD presents with a much wider range of cancer types, particularly brain tumors, hematological malignancies and colorectal cancer, which mostly develop in childhood. Whereas lymphomas comprise ~ 15% of cancers in CMMRD, these are mainly T-cell lymphomas and reports of DLBCL are less frequent [15]. The patient we report here had no additional clinical features of CMMRD, nor did we find a second pathogenic germline or somatic variant in *MSH6*. The tumor did not show signs of hypermutation or a mismatch repair associated mutation spectrum. Furthermore, all MMR markers were expressed normally, and microsatellite instability was absent. Hence, we could not confirm a causal relation between loss of the *MSH6* allele and development of the DLBCL. Because of the germline deletion of *MSH6* the patient was diagnosed with Lynch syndrome, and colorectal cancer surveillance was recommended from the age of 25 years onwards.

Since the first description of a patient with a *FBXO11* alteration and developmental delay in 2016, a total of 71 patients have been published [10, 11, 16–25]. Forty single nucleotide variants and indels were detected in 46 patients, including 18 missense, 5 nonsense, 10 frameshifts, 5 splice site variants, and 2 in-frame deletions [10, 11, 16–19]. All variants were de novo, except for one frameshift variant, which has recently been described in two sisters with developmental delay who inherited the variant from their mother [19]. In addition, 25 individuals have been described with a germline partial gene deletion of *FBXO11* or a larger deletion including the *FBXO11* gene [10, 11, 20–26]. Of these individuals, seven patients carry a deletion limited to the *FBXO11* and *MSH6* genes and four patients carry a partial deletion of *FBXO11* only. Larger deletions often include the adjacent *MSH6* and *MSH2* genes. Common phenotypic presentations for all patients are developmental delay and behavior abnormality including autism, hyperactivity, and anxiety. Two of these patients, both affected by a large deletion in chromosome 2p16.3-p21 affecting multiple genes including *FBXO11, MSH6* and *MSH2*, developed a malignancy. A male patient developed a prostate adenocarcinoma and a synchronous sigmoid adenocarcinoma at the age of 52. Both tumors showed loss of MSH2 and MSH6 expression [25]. A female patient developed a mucinous adenocarcinoma of the colon with loss of MSH2 [22]. The tumors in both patients were attributed to Lynch syndrome. No other malignancies have been reported in patients with a *FBXO11* alteration.

So far, germline aberrations in *FBXO11* have not been linked to cancer development. However, a relation between somatic *FBXO11* alterations and cancer has been described. FBXO11 is part of the F-Box protein family, of which the members are the substrate recognition subunits of the SKPI-Cullin1-F-box (SCF) complex [27]. This complex is responsible for the catalyzation of ubiquitination and subsequent proteasomal degradation of substrates [28]. The SCF mediated process of protein degradation is important for genome stability and maintenance [29]. According to the cBioPortal and COSMIC databases, *FBXO11* alterations have been detected in various adult neoplasms, including colon cancer, lung cancer, ovarian cancer, and head and neck cancer, as well as in diffuse large B-cell lymphoma (DLBCL) [30–32]. Recently, *FBXO11* was also identified as a potential tumor suppressor in myelodysplastic syndrome and secondary acute myeloid leukemia [33, 34]. In approximately 4–8% of adult patients with DLBCL a somatic genetic alteration of the *FBXO11* gene is present [35, 36]. According to the St. Jude Cloud PeCan database, somatic *FBXO11* variants have been detected in eight pediatric oncology patients of whom seven were diagnosed with a Burkitt lymphoma (BL), but none with a DLBCL [37]. These variants include four missense variants, two frameshift variants located in the Beta Helix domain, and a stop-loss variant causing a 16-nucleotide extension of the open reading frame. Although DLBCL and BL are classified as two different types of lymphoma, similarities on both morphological and molecular level can make it difficult to distinguish them [38, 39].

Patients with DLBCL frequently carry somatic mutations in *BCL6*, a known proto-oncogene. BCL-6 is present in the germinal center and withholds premature B-cell activation and differentiation into mature plasma cells and memory B-cells [40]. BCL-6 expression is retained in most patients with DLBCL [41]. One of the mechanisms, resulting in retained BCL-6 expression is insufficient proteasomal degradation, which occurs as a result of diminished ubiquitination of BCL-6 by the mutated FBXO11 protein [35, 36]. Duan et al. found monoallelic deletions and mutations of *FBXO11* in DLBCL, and suggested that *FBXO11* is a haplo-insufficient tumor suppressor gene [35]. We hypothesize that the heterozygous germline *FBXO11* deletion in the patient would result in retained BCL-6 expression in the tumor. However, immunohistochemistry of the tumor showed negative staining for BCL-6. No somatic aberration of *BCL6* was discovered that could explain this phenomenon.

Based on the role of FBXO11 in DLBCL, we consider that the germline heterozygous *FBXO11* deletion may have contributed to the DLBCL development in this child, although we cannot exclude that the development of DLBCL may be a coincidence. Thus far, no cancer attributed to the *FBXO11* aberration has been reported in a total of 71 patients with germline *FBXO11* alterations. This lack of cancer might be explained by reduced penetrance, as is the case for many other childhood cancer predisposing genes [42], and by the relatively low age of these patients at time of publication (median age of 9.25 years in a cohort of 61 individuals). Therefore, assessment of larger cohorts of individuals with constitutional *FBXO11* aberrations is required to establish whether the incidence of lymphoma is indeed enriched in these individuals.

## Conclusion

In conclusion we present a case of a boy with developmental delay resulting from a de novo germline 2p16.3 deletion including *FBXO11* and *MSH6*, who developed a diffuse large B-cell lymphoma. We found no evidence of MSH6 inactivation nor MRR deficiency in the tumor, hence a causative role for the germline deletion of *MSH6* in the development of the lymphoma was excluded. We consider a causative relationship with the germline deletion of *FBXO11*, a haplo-insufficient tumor suppressor in this cancer type, conceivable.

## Supplementary Information

Below is the link to the electronic supplementary material.

Electronic supplementary material 1 (DOCX 29 kb)

## Data Availability

Data is available upon request.
