# Supplementary material for: Constitutional 2p16.3 deletion including MSH6 and FBXO11 in a boy with developmental delay and diffuse large B-cell lymphoma
Source: Fam Cancer. 2021 Apr 3;20(4):349–54. doi: 10.1007/s10689-021-00244-2 (PMC8484184; doi:10.1007/s10689-021-00244-2)
Supplement: Supplementary file 1 — Electronic supplementary material 1 (DOCX 29 kb) [file 10689_2021_244_MOESM1_ESM.docx]

**Supplementary data**

**Table 1: Somatic variants derived by whole exome sequencing**

| Gene | Position (GRCh38) | Transcript | cDNA | Amino Acid change | Coding effect | VAF (%) | CADD | SIFT | PolyPhen-2 | PhyloP | ADA | pLi |
| --- | --- | --- | --- | --- | --- | --- | --- | --- | --- | --- | --- | --- |
| *TP53* | Chr17: 7674221G>A | NM_00546.6 | c.742C>T | p.(Arg248Trp) | M | 45.7 | 26.6 | D | D | 0.835 |  | 0.53 |
| *EEF1D* | Chr8:  143586290C>A | NM_032378.7 | c.1216G>T | p.(Glu406*) | N | 36.6 | 47 | - | - | 5.773 | 0,999 | 8.99x10^-7 |
| *NALCN* | Chr13: 101074652T<G | NM_052867.4 | c.3965A>C | p.(Lys1322Thr | M | 30.9 | 26.6 | D | D | 7.674 |  | 8.52x10^-14 |
| *MYC* | Chr8: 127738438C>T | NM_002467.6 | c.221C>T | p.(Pro74Leu) | M | 29.5 | 33 | D | D | 7.884 |  | 0.99 |
| *PTPRG* | Chr3: 62168100G>T | NM_002841.4 | c.970G>T | p.(Asp324Tyr) | M | 26.7 | 25.7 | D | D | 7.680 |  | 5.07x10^-6 |
| *NALCN* | Chr13: 101074549T>A | NM_052867.4 | c.4068A>T | p.(Leu1356Phe) | M | 26.5 | 23.3 | D | D | -0.066 |  | 8.52x10^-14 |
| *NBEA* | Chr13: 35156110T>TA | NM_015678.4 | c.2555-2556insA | p.(Asn854fs*18) | F | 25.0 | - | - | - |  |  | 1.00 |
| *GPLD1* | Chr6: 24467243A>T | NM_001503.4 | c.577T>A | p.(Tyr193Asn) | M | 24.2 | 26 | D | D | 6.283 |  | 1.59x10^-15 |
| *TFAP4* | Chr16: 4262618C>T | NM_003223.3 | c.173G>A | p.(Arg58Gln) | M | 24.1 | 28.6 | D | D | 7.292 |  | 0.99 |
| *HIST1H2BG* | Chr6: 26216533G>C | NM_003518.3 | c.111C>G | p.(Ser37Arg) | M | 23.8 | 25.8 | D | - | 0.610 |  | 4.89x10-7 |
| *MYC^1^* | Chr8: 127738923G>C | NM_002467.6 | c.706G>C | p.(Asp236His) | M | 23.8 | 19.46 | D | D | 2.980 |  | 0.99 |
| *MYC^1^* | Chr8: 127738945A>T | NM_002467.6 | c.728A>T | p.(Glu243Val) | M | 23.7 | 19.87 | D | B | 0.554 |  | 0.99 |
| *SYT11* | Chr1: 155868681C>T | NM_152280.5 | c.751C>T | p.(Arg251Cys) | M | 22.7 | 29 | D | D | 6.024 |  | 0.93 |
| *EIF5B* | Chr2: 99363864T>C | NM_015904.4 | c.1137+2T>C |  | S | 22.6 | 32 | - | - | 6.702 | 0,992 | 1.00 |
| *DDX46* | Chr5: 134794859A>C | NM_014829.4 | c.1636A>C | p.(Ile546Leu) | M | 22.3 | 24.6 | D | P | 9.175 |  | 1.00 |
| *ZNF484* | Chr9: 92847664T>A | NM_031486.4 | c.1123A>T | p.(Lys375*) | N | 21.4 | 35 | - | - | -0.128 |  | 1.16x10^-8 |
| *BNC2* | Chr9: 16727883C>T | NM_017637.6 | c.244G>A | p.(Gly81Arg) | M | 20.4 | 24.5 | D | P | 5.401 |  | 0.93 |
| *CASP8* | Chr2: 201266782C>T | NM_001228.4 | c.296C>T | p.(Ser99Phe) | M | 18.6 | 25.3 | D | D | 4.244 |  | 3.76x10^-6 |
| *CXCR4* | Chr2: 136115848-136115858del | NM_003467.3 | c.70_80del | p.(Met24Leufs*4) | F | 18.0 |  |  |  |  |  | 0.02 |
| *CCND3* | Chr6: 41936008dup | NM_001760.5 | c.811dup | p.(Arg271Profs*53) | F | 17.1 |  |  |  |  |  | 0.98 |
| *ITPR3* | Chr6: 33655795C>T | NM_002224.4 | c.190C>T | p.(Arg64Cys) | M | 15.4 | 24.2 | D | D | 1.722 |  | 2.55x10^-22 |
| *ADK* | Chr10: 74670244T>G | NM_006721.4 | c.939T>G | p.(Asn313Lys) | M | 10.9 | 24 | D | D | 1.386 |  | 3.13x10^-4 |

The sequence data was aligned to genome build GRCh38 using the GATK best practices. Somatic variant calling was performed using GATK Mutect2 and annotated the variant according to the HGVS nomenclature.[43]

^1^Both variants are located on the same strand

VAF= Variant allele frequency; ADA [44]; pLi= expected Probability of loss of function intolerance [45]; M= Missense; N= Nonsense; F= Frameshift; S= Splice site; D= Damaging; B= Benign; P= Probably damaging
